# Comparison of online and face-to-face valuation of the EQ-5D-5L using composite time trade-off

**DOI:** 10.1007/s11136-020-02712-1

**Published:** 2020-11-28

**Authors:** Ruixuan Jiang, James Shaw, Axel Mühlbacher, Todd A. Lee, Surrey Walton, Thomas Kohlmann, Richard Norman, A. Simon Pickard

**Affiliations:** 1 Center for Observational and Real-World Evidence, Merck, Kenilworth, NJ USA; 2 Patient-Reported Outcomes Assessment, Bristol-Myers Squibb, Princeton, NJ USA; 3 Health Economics and Healthcare Management, Hochschule Neubrandenburg, Neubrandenburg, Germany; 4 Department of Pharmacy Systems, Outcomes, and Policy, University of Illinois At Chicago College of Pharmacy, Chicago, IL USA; 5 Institute for Community Medicine, Medical University Greifswald, Greifswald, Germany; 6 Faculty of Health Sciences, Curtin University School of Public Health, Perth, Australia

**Keywords:** Time trade-off, EQ-5D, Preference elicitation, Online, Face-to-face

## Abstract

**Objective:**

The aim of this study was to compare online, unsupervised and face-to-face (F2F), supervised valuation of EQ-5D-5L health states using composite time trade-off (cTTO) tasks.

**Methods:**

The official EuroQol experimental design and valuation protocol for the EQ-5D-5L of 86 health states were implemented in interviewer-assisted, F2F and unsupervised, online studies. Validity of preferences was assessed using prevalence of inconsistent valuations and expected patterns of TTO values. Respondent task engagement was measured using number of trade-offs and time per task. Trading patterns such as better-than-dead only was compared between modes. Value sets were generated using linear regression with a random intercept (RILR). Value set characteristics such as range of scale and dimension ranking were evaluated between modes.

**Results:**

Five hundred one online and 1,134 F2F respondents completed the surveys. Mean elicited TTO values were higher online than F2F when compared by health state severity. Compared to F2F, a larger proportion of online respondents did not assign the poorest EQ-5D-5L health state (i.e., 55555) the lowest TTO value ([Online] 41.3% [F2F] 12.2%) (*p* < 0.001). A higher percentage of online cTTO tasks were completed in 3 trade-offs or fewer ([Online] 15.8% [F2F] 3.7%), (*p* < 0.001). When modeled using the RILR, the F2F range of scale was larger than online ([Online] 0.600 [F2F] 1.307) and the respective dimension rankings differed.

**Conclusions:**

Compared to F2F data, TTO tasks conducted online had more inconsistencies and decreased engagement, which contributed to compromised data quality. This study illustrates the challenges of conducting online valuation studies using the TTO approach.

**Electronic supplementary material:**

The online version of this article (10.1007/s11136-020-02712-1) contains supplementary material, which is available to authorized users.

## Introduction

Valuation studies of measures of health, e.g., the EQ-5D, are traditionally conducted in-person with trained interviewers. This face-to-face elicitation of preferences has been refined and may be considered the de facto standard to ensure respondent attendance/engagement with an understanding of the task. However, such a process is resource and time-intensive [1–4]. Additional shortcomings of in-person studies include potential social desirability bias in respondent answers, difficulty recruiting certain populations, and respondent unwillingness to answer sensitive questions, which may contribute to missing data, increase bias, and limit generalizability [3, 5]. Online data collection, typically using panels, has recently increased in popularity and has several advantages over in-person methods, including greater geographic reach, additional respondent convenience, lower study cost, and more rapid data collection [4]. However, selection biases also exist online, although different from those which affect in-person data collection (e.g., differential internet access among segments of the population) [4, 6].

Problematically, certain types of preference elicitation techniques, such as the time trade-off (TTO), may not lend themselves well to online, unsupervised data collection due to the complexity and iterative nature of the task [3]. Interviewer assistance is often needed to ensure task comprehension and allow for interactive task clarification in real-time. For example, the validity of responses can be compromised if respondents do not understand the TTO or shortcut tasks. Challengingly, without assured task comprehension and engagement, inclusion and exclusion of observations when estimating a value set can be subjective, which may add uncertainty to and/or shift the utility estimates [7–10]. Further, exclusion of observations diminishes the sample size and may affect the generalizability of the final value set.

With advancing technology and increasing access to the internet, online data collection is likely to grow in popularity [4]. Thus, there is a need to evaluate differences between preferences collected using online and in-person modes [11]. This knowledge can inform greater understanding of variation between value sets if the valuation studies were conducted using different modes. Further, this understanding can contribute to critical appraisal of cost-utility analyses by clarifying the origins of health valuations [11, 12].

To date, few studies have compared the quality and validity of different modes of data collection for a given preference elicitation technique. In this work, the evidence-based, internationally standardized EQ-5D-5L valuation study protocol was conducted both face-to-face and online, presenting an opportunity to investigate if the traditional and newer modes of data collection are able to produce the same results. The goal of this study was to determine whether the in-person, interviewer-supervised composite time trade-off (cTTO) results were replicable in an online, unsupervised respondent group.

## Methods

### Data sources and measure

The EQ-5D is a generic measure of health used in a variety of applications [13, 14]. The EQ-5D-5L is composed of 5 dimensions of health: Mobility, Self-Care, Usual Activities, Pain/Discomfort, and Anxiety/Depression, and 5 levels of severity: no, mild, moderate, severe, and extreme problems (unable to) on a given dimension [15–17]. It describes 3,125 health states ranging from 11111 (no problems on any dimension) to 55555 (extreme problems or unable to on all dimensions). A misery score can also be calculated by summing the numbers of the 5-digit health state string to approximate health state severity [18].

This study harnessed data from the US EQ-5D-5L face-to-face (F2F) valuation study and the US EQ-5D-5L online valuation experimental study [19]. The F2F study used the EuroQol Valuation Technology (EQ-VT), a standardized platform developed by the EuroQol group and implemented the most recent version of the valuation protocol, version 2.0 [20]. The online study was conducted by SurveyEngine, a company specializing in preference elicitation, and used an online platform modeled after the EQ-VT. Online platform designers and researchers involved in both face-to-face and online studies ensured platform equivalence (Appendix A). Some interviewer cues and tutorials were altered to optimize the study for online administration and simulate the role of an interviewer based on assessor feedback (Appendix B).

### Experimental design and preference elicitation task

The experimental design (i.e., health states valued and their blocking) was identical in the online and face-to-face studies [21]. The experimental design was made up of 86 EQ-5D-5L health states. It included the 5 mildest, suboptimal EQ-5D-5L health states (i.e., slight problems on a single dimension; misery score 6), the poorest EQ-5D-5L health state (55555; misery score 25), and 80 other health states [22]. Each of the 10 TTO blocks included a mild health state, 55555, and 8 additional health states. Each respondent was randomly assigned to a TTO block, and the health states were presented in random order.

Both the online and face-to-face studies used the composite time trade-off (cTTO) to elicit preferences on a cardinal scale [23]. The cTTO began with the conventional TTO to elicit better-than-dead (BTD) TTO values, and 10 years in the suboptimal health state being valued (Life B) was compared to 10 years in Full Health (Life A). The lead-time time trade-off (LT-TTO) was used to elicit worse-than-dead (WTD) TTO values, and the respondent was provided with 10 additional years in Full Health in both Life A and Life B to trade. In each TTO task subtype, time in Life A changed according to an automated ping-pong/titration process (Appendix C) until the respondent felt that Life A and Life B were approximately the same. [20]

### Data collection methods, survey platforms, and comparator groups

All respondents were quota-sampled for age, gender, race, and ethnicity according to the most recent official estimates of the US general adult population.

#### Face-to-face study

Face-to-face respondents were recruited from a variety of sources, including in-person recruitment and advertising to online forums [19]. Computer-assisted personal interviews (CAPI) were conducted one-on-one between the interviewer and the respondent in centralized city and suburban locations throughout 6 US metropolitan areas. Face-to-face respondents were paid $30 cash for the interview.

For all tasks, respondents read each health state aloud and were encouraged to think aloud so interviewers could detect and react to misunderstandings. Five practice health states were presented to familiarize the respondent with the cTTO and the EQ-5D-5L [20]. The first two examples used life in a wheelchair as the reference point for suboptimal health states to familiarize respondents with the conventional (BTD) and LT-TTO (WTD) preference elicitation. Three EQ-5D-5L health states then followed, in order as follows: mild, severe, and “implausible” health states. The mild and severe health states demonstrated the severity range of health states to be valued. Two dimension-levels in the “implausible” health state appeared unlikely to co-occur, but the combination was plausible once explained. It was used to emphasize that the respondent should try to envision each health state presented. The values respondents provided on practice health states were not included in the value set modeling or any other analysis.

Ten EQ-5D-5L cTTO tasks followed the practice tasks [20]. After these tasks, the EQ-5D-5L health states were sorted by respondent-assigned TTO values in the feedback module. Respondents reviewed their response to each health state. If the respondent found that a health state was valued incorrectly, that health state could be marked and removed from analyses.

Two main F2F comparator groups were created: (1) all F2F respondents and their complete cTTO-elicited preferences (F2F Full; F2F_F_) (2) F2F respondents who understood the cTTO task per interviewer judgment excluding those responses that respondents themselves flagged in the feedback module (F2F Valid; F2F_V_). F2F Full represented complete respondent preferences without any additional interviewer or respondent judgements on preference validity. The F2F Valid sample represented the most valid preferences following elimination of both interviewer- and respondent-judged invalid responses. The interviewer judged invalid F2F respondents (F2F Invalid; F2F_I_) were used in exploratory, post hoc analyses.

#### Online experimental study

Online respondents were recruited from panels and paid per survey in credit equivalent to a few dollars according to usual practice. For all online tasks, the health state was displayed on the page prior to the task, presented word by word, and read aloud by an automated female American voice (Appendix D). Respondents could not proceed until the reading was completed. The same five practice tasks were implemented in the online and F2F studies in the same order. Respondents learned the conventional and LT-TTO tasks to indicate BTD and WTD values, respectively, in an interactive tutorial using life in a wheelchair as the suboptimal health state (Appendix E). Respondents were required to perform specific actions, such as “click the B button until Life A is at 7.5 years”, in order to move onto the next tutorial step. The tutorial could be repeated.

The EQ-5D-5L practice health states were not framed as training tasks to minimize respondent frustration. However, additional instructions were provided with the implausible EQ-5D-5L health state to remind respondents to envision every health state being valued, even if they seemed unlikely to exist. As in the face-to-face arm, the five practice tasks were not included in any analysis. If a task was completed in less than 15 s, a pop-up box was displayed to induce more careful consideration to parallel the interviewer’s efforts in the F2F survey. The feedback module was removed from the online study as it was too difficult to explain to the respondent without an interviewer. All online respondents who completed the survey and their preferences were included in the Online comparator.

### Data analyses

#### Descriptive statistics

Face validity of the data was assessed using the distribution of the TTO values and means and standard deviations of the elicited TTO values by misery score. For adequate face validity, the TTO value means should decrease and the standard deviations are likely to increase with worsening health states (i.e., increasing misery scores). This pattern represents a lessening desirability and rising disagreement with the valuation of more severe health states. Face validity was also separately assessed for the first five and last five presented TTO tasks to evaluate if either mode of administration was subject to an order effect. The TTO values from the subset of online respondents who report agreement or strong agreement with the TTO being easy to understand were also analyzed to evaluate whether face validity was greater as compared to the overall online sample.

Preference patterns were constructed from the TTO values to characterize trading and compare respondent engagement and lower-quality preferences between arms. Trading patterns evaluated included BTD-only traders (all TTO value > 0) and non-traders (all TTO values = 1). Respondent engagement was assessed by the prevalence of low engagement trading (all TTO tasks completed with 3 trade-offs or fewer), time per task, and mean number of trade-offs per task. Data validity was measured using the proportion of respondents with at least 1 inconsistency (higher TTO value assigned to dominated health state) and at least 1 inconsistency involving the worst EQ-5D-5L health state as well as the mean number of these inconsistencies per respondent. The number and magnitude of inconsistencies were examined as a function of the misery score difference between health state pairs. Fewer inconsistencies were expected when misery score difference was large.

#### Modeled value sets

Each set of TTO data was modeled using a linear regression with a respondent-level random intercept (RILR). The dependent variable was elicited TTO values, and the independent variables were dummy variables for decrements from level 1 (no problems) on each dimension. The number of preference inversions (i.e., the disutility for a level was less negative than a milder level within the same dimension), relative importance of dimensions, percentage of modeled health states that were WTD, and range of scale were visually compared between online and face-to-face comparators. The effect of mode of administration was estimated using a dummy variable. This RILR was estimated over both F2F and online responses (unadjusted analyses). Respondent characteristics hypothesized to affect respondent valuations were included as covariates in adjusted analyses [24]. These factors included age, gender, race, ethnicity, US census region, self-reported TTO comprehension, general health, experience with serious illness, education, and health literacy [25]. The odds ratio of providing at least 1 inconsistent valuation by online respondents was assessed using logistic regression, and time spent on the TTO tasks as well as the covariates used in adjusted RILR analyses were included for adjustment in the logistic regression.

## Results

### Respondents

One thousand one hundred and thirty-four respondents completed the face-to-face survey with 11,340 responses (F2F Full; F2F_F_), while 501 respondents completed the online survey with 5010 responses (Online) (Table 1; Appendix F). Interviewers determined that 72 F2F respondents did not understand the TTO (F2F Invalid; F2F_I_). The remaining 1062 F2F respondents retracted 1234 TTO valuations in the feedback module, leaving the F2F Valid (F2F_V_) sample with 9386 responses. Both main F2F comparators and the Online sample were generally similar to the US adult general population (data not shown).

**Table 1:** Respondent characteristics

Characteristic	(1) F2F full sample (n = 1,134)	(2) F2F valid sample (n = 1,062)	(3) Online (n = 501)	(1) vs. (3) p-value	(2) vs. (3) p-value
Age, mean (SD), n (%)	46.9 (18.1)	46.9 (18.0)	45.9 (15.1)	0.25	0.28
18–34	358 (31.6)	347 (32.7)	149 (29.7)	0.76	0.51
35–54	394 (34.7)	365 (34.4)	180 (35.9)		
55 +	382 (33.7)	350 (33.0)	172 (34.3)		
Range	18–99	18–99	17–80		
Gender, n (%)					
Male	564 (49.7)	515 (48.5)	251 (50.1)	0.33	0.27
Female	565 (49.8)	542 (51.0)	250 (49.9)		
Gender, other	5 (0.4)	5 (0.5)	–		
Race, n (%)					
White	685 (60.4)	661 (62.2)	387 (77.3)	0.65	0.77
Black	152 (13.4)	128 (12.1)	63 (12.6)		
Hispanic ethnicity, n (%)	208 (18.3)	191 (18.0)	75 (15.0)	0.10	0.14
Education level greater than secondary, n (%)	732 (64.6)	703 (66.2)	344 (68.7)	0.11	0.33
Child dependents					
None	916 (80.8)	857 (80.8)	338 (67.5)	0.01	0.01
Child(ren), ≤ 5 years old	68 (6.0)	65 (6.1)	65 (13.0)	< 0.0001	< 0.0001
Child(ren), 6 to 17 years old	180 (15.9)	169 (15.9)	138 (27.5)	< 0.0001	< 0.0001
Primary health insurance					
None	98 (8.6)	89 (8.4)	49 (9.8)	0.69	0.67
Public	480 (42.3)	434 (40.9)	204 (40.5)		
Private	555 (49.1)	538 (50.7)	249 (49.7)		
Country of birth, United States	983 (86.7)	929 (87.6)	475 (94.8)	< 0.0001	< 0.0001
History of illness, n (%)					
Hypertension	270 (23.8)	245 (23.1)	141 (28.1)	0.06	0.03
Arthritis	267 (23.5)	244 (23.0)	120 (24.0)	0.86	0.67
Diabetes	111 (9.8)	95 (9.0)	71 (14.2)	0.009	0.002
Heart Failure	20 (1.8)	18 (1.7)	11 (2.2)	0.54	0.62
Stroke	23 (2.0)	20 (1.9)	11 (2.2)	0.83	0.68
Bronchitis	29 (2.6)	23 (2.2)	18 (3.6)	0.25	0.1
Asthma	132 (11.6)	125 (11.8)	52 (10.4)	0.46	0.42
Depression	295 (26.0)	270 (25.5)	117 (23.4)	0.25	0.38
Migraine	164 (14.5)	154 (14.5)	58 (11.6)	0.12	0.12
Cancer	65 (5.7)	59 (5.6)	12 (2.4)	0.003	0.005
None	372 (32.8)	356 (33.6)	157 (31.3)	0.56	0.39
Health status, n (%) (44)					
Excellent / Very good / Good	980 (86.4)	923 (86.9)	411 (82.0)	0.02	0.01
Fair / Poor	154 (13.5)	139 (13.1)	90 (18.0)		
Self-reported EQ-VAS					
Mean (SD)	80.4 (15.6)	80.5 (15.5)	73.6 (20.4)	< 0.0001	< 0.0001
Median (IQR)	85 (15)	85 (15)	80 (25)		
"I found it easy to understand the questions I was asked"					
Strongly agree	596 (52.6)	585 (53.1)	239 (47.7)	< 0.0001	< 0.0001
Agree	445 (39.2)	437 (39.7)	166 (33.1)		
Neither agree nor disagree	49 (4.3)	42 (3.8)	50 (10.0)		
Disagree	39 (3.4)	36 (3.3)	34 (6.8)		
Strongly disagree	5 (0.4)	2 (0.2)	12 (2.4)		
“I found it easy to tell the difference between the lives I was asked to think about”					
Strongly agree	540 (47.6)	526 (47.7)	214 (42.7)	< 0.0001	< 0.0001
Agree	460 (40.6)	453 (41.1)	185 (36.9)		
Neither agree nor disagree	75 (6.6)	68 (6.2)	60 (12.0)		
Disagree	53 (4.7)	51 (4.6)	32 (6.4)		
Strongly disagree	6 (0.5)	4 (0.4)	10 (2.0)		
"I found it difficult to decide on my answers to the questions"					
Strongly agree	207 (18.3)	196 (17.8)	90 (18.0)	< 0.0001	< 0.0001
Agree	433 (38.2)	424 (38.5)	144 (28.7)		
Neither agree nor disagree	164 (14.5)	156 (14.2)	107 (21.4)		
Disagree	207 (18.3)	205 (18.6)	92 (18.4)		
Strongly disagree	123 (10.9)	121 (11.0)	68 (13.6)		

F2F face-to-face

Quota-sampled characteristics, education attainment, and insurance coverage type were similar between Online, F2F_F_, and F2F_V_ samples (Table 1). Online respondents tended to be less healthy, with lower mean values on the visual analog scale (VAS) and poorer general health (*p* < 0.0001, *p* < 0.02, respectively). Online respondents were also more likely to have children under 18 and report problems with TTO comprehension (*p* < 0.0001).

### Raw elicited TTO values and preference patterns

**Fig. 1:**
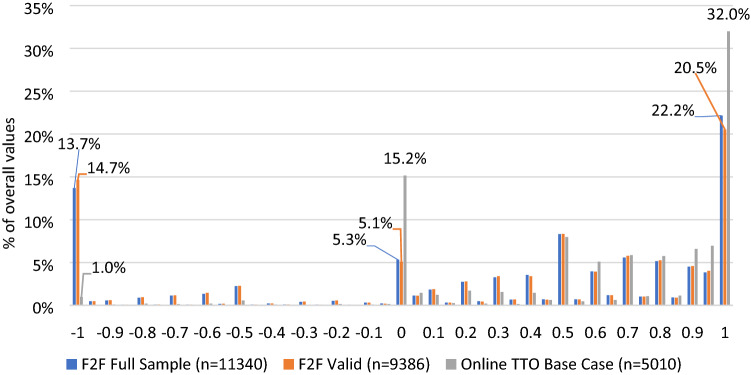
Distribution of time trade-off values by mode of administration. *F2F* face-to-face, *TTO* time trade-off

Raw TTO values differed between Online and main F2F comparators. Online respondents provided fewer WTD TTO values: [Online] 2.8%; [F2F_F_] 22.7%; [F2F_V_] 24.0% (Fig. 1). The proportions of tasks which accessed the LT-TTO/WTD section were similar between F2F_F_, F2F_V,_ and online (23.2–24.2%). However, conversion to WTD values was less likely in online compared to F2F: [F2F_V_] 93.8% [F2F_F_] 94.6% [Online] 37.1% (Appendix H). Online responses showed larger local maximums (“spikes”) at 0 and 1 and smaller spike at -1 compared to F2F_F_ and F2F_V_. Compared to Online, F2F_I_ responses yielded a larger spike at 1, but patterns of mean and standard deviations of TTO values were similar (Online Appendix I, J).

**Fig. 2:**
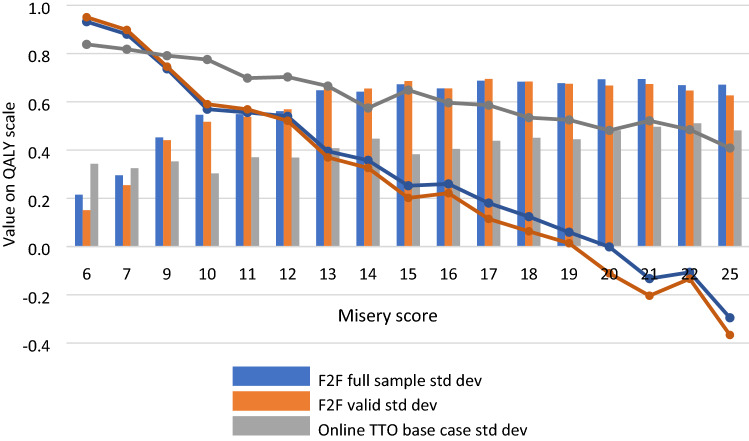
Mean and standard deviations of time trade-off values by misery score. *F2F* face-to-face, *TTO* time trade-off, *std dev* standard deviation

Mean elicited TTO values decreased with increasing health state misery score in both main F2F comparators and the online arm (Fig. 2). Compared to F2F_F_ and F2F_V_, Online mean TTO values were lower for milder health states (misery score 6 and 7) but higher for all other health states. For the F2F arms, the standard deviations increased at a faster rate, whereas the online standard deviations remained comparatively constant (Fig. 2), potentially indicating similar rates of disagreement throughout the range of health states valued in online respondents. Face validity for the modeled values of the first five and last five TTO tasks appeared similar within each mode of administration (data not shown). The online respondents who reported agreement/strong agreement with the TTO task being easy to understand did not provide different TTO values than those who did not (*p*-value = 0.961; data not shown).

Compared to both main F2F comparators, online respondents were more likely to be BTD-only traders (F2F_F_ 31.0%; F2F_V_ 12.7%; Online 46.3%; *p*-values < 0.0001), non-traders (F2F_F_ 5.7%; F2F_V_ 3.0%; Online 7.2%; *p*-values < 0.0001), and use 3 trade-offs or fewer to complete all tasks (F2F_F_ 3.7%; F2F_V_ 1.6%; Online 15.8%; *p*-values < 0.0001)(Table 2). Online respondents also used fewer trade-offs per task on average (F2F_F_ 6.6 (SD 4.8); F2F_V_ 6.7 (4.6); Online 5.6 (5.8) *p*-values < 0.0001). Descriptive analysis of TTO task characteristics (number of moves, time, etc.) stratified by trading behavior did not reveal consistent patterns to support validity of online responses. (Online Appendix K).

**Table 2:** Respondent engagement and data validity summary

Level		(1) F2F Full		(2) F2F valid		(3) Online		(1) vs (3) p-value	(2) vs (3) p-value
Task		N = 11,340		N = 9,386		N = 5,010			
		Mean	SD	Mean	SD	Mean	SD		
	Trade-offs	6.6	4.8	6.7	4.6	5.6	5.8	< 0.0001	< 0.0001
	TTO value	0.32	0.69	0.30	0.71	0.63	0.43	< 0.0001	< 0.0001
		Median	IQR	Median	IQR	Median	IQR		
	Time per task in seconds	49.75	31.22—80.83	49.75	31.90—79.93	46.75	36.71—63.61		
Respondent		N = 1,134		N = 1,062		N = 501			
		N	%	N	%	N	%		
	Better-than-dead-only traders	351	31.0%	135	12.7%	232	46.3%	< 0.0001	< 0.0001
	Non-traders (All TTO values = 1)	65	5.7%	32	3.0%	36	7.2%	< 0.0001	< 0.0001
	All tasks completed within 3 trade-offs	42	3.7%	17	1.6%	79	15.8%	< 0.0001	< 0.0001

*F2F* face-to-face, *TTO* time trade-off, *IQR* interquartile range

**Fig. 3:**
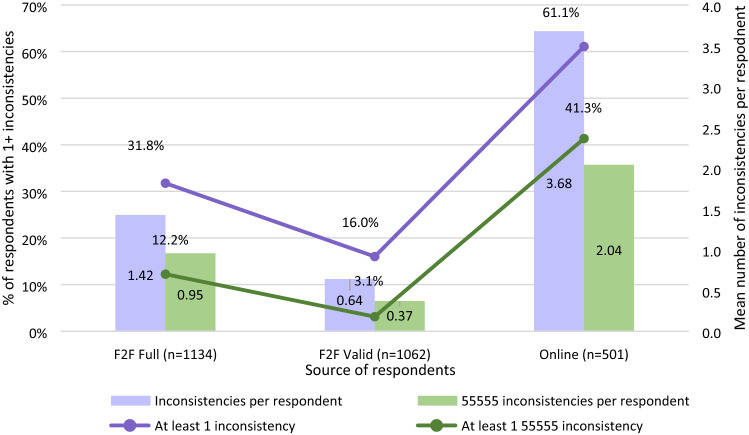
Mean number of inconsistencies per respondent and prevalence of inconsistencies. *pF2F* face-to-face, *TTO* time trade-off, *std dev* standard deviation

Online respondents were more likely to commit at least 1 inconsistency (any and involving 55555). Over 60% and 40% of online respondents had at least 1 inconsistency of any kind and 55555-involved, respectively, whereas corresponding numbers for F2F_F_/F2F_V_ were 16.0%/31.8% and 3.1%/12.2% (Fig. 3). Online respondents also had higher mean number of inconsistencies per respondent compared to F2F_F_ and F2F_V_. On average, online respondents had more than 2–5 times the number of inconsistencies as the F2F Full and F2F Valid samples, respectively (Fig. 3). Online respondents invariably were 2–3 times more likely to produce at least 1 inconsistency no matter the sequence of the 55555 presentation (Appendix L). Online respondents provided both larger TTO inconsistencies and more inconsistencies than F2F comparators (Online Appendix M).

### Modeled value sets

The decrement for each dimension level of the online value set was smaller than the same decrement for the F2F Full or Valid samples (Table 3). All estimated parameters for F2F_F_ and F2F_V_ were significantly different from the reference level of “no problems”, whereas the Online value set had 8 insignificant parameters. Additionally, the main F2F comparators each had a single, significant preference inversion (UA5), while the Online sample had two (UA4 and SC5). The F2F_I_ modeled value set had 17 insignificant parameters and 8 preference inversions.

**Table 3:** Modeled value sets for Face-to-Face Full, Face-to-Face Valid, and Online comparators

	F2F Full (Full Sample) cTTO			F2F Valid (interviewer judged valid; feedback module applied) cTTO			Online cTTO		
	Estimate	SE	p-value	Estimate	SE	p-value	Estimate	SE	p-value
Intercept	0.963	0.020	< .0001	0.993	0.02051	< .0001	0.846	0.021	< .0001
MO2	− 0.085	0.013	< .0001	− 0.089	0.015	< .0001	− 0.026	0.016	0.114^
MO3	− 0.123	0.014	< .0001	− 0.128	0.015	< .0001	− 0.043	0.017	0.011
MO4	− 0.199	0.015	< .0001	− 0.224	0.017	< .0001	− 0.067	0.019	0.000
MO5	− 0.253	0.014	< .0001	− 0.288	0.015	< .0001	− 0.112	0.017	< .0001
SC2	− 0.067	0.013	< .0001	− 0.080	0.014	< .0001	− 0.003	0.016	0.874^
SC3	− 0.096	0.015	< .0001	− 0.111	0.016	< .0001	− 0.035	0.018	0.055^
SC4	− 0.181	0.015	< .0001	− 0.208	0.016	< .0001	− 0.098	0.018	< .0001
SC5	− 0.213	0.013	< .0001	− 0.231	0.015	< .0001	− 0.077	0.016	< .0001*
UA2	− 0.056	0.014	< .0001	− 0.060	0.015	< .0001	− 0.030	0.017	0.075^
UA3	− 0.090	0.015	< .0001	− 0.100	0.016	< .0001	− 0.067	0.018	0.000
UA4	− 0.218	0.015	< .0001	− 0.240	0.016	< .0001	− 0.059	0.018	0.001*
UA5	− 0.188	0.014	< .0001	− 0.217	0.015	< .0001	− 0.075	0.016	< .0001
PD2	− 0.057	0.013	< .0001	− 0.069	0.013	< .0001	− 0.020	0.015	0.187^
PD3	− 0.094	0.015	< .0001	− 0.103	0.016	< .0001	− 0.023	0.018	0.210^
PD4	− 0.268	0.013	< .0001	− 0.296	0.015	< .0001	− 0.090	0.016	< .0001
PD5	− 0.333	0.015	< .0001	− 0.364	0.016	< .0001	− 0.108	0.018	< .0001
AD2	− 0.049	0.015	0.001	− 0.050	0.016	0.001	− 0.010	0.018	0.586^
AD3	− 0.118	0.016	< .0001	− 0.128	0.018	< .0001	− 0.031	0.020	0.114^
AD4	− 0.271	0.015	< .0001	− 0.288	0.016	< .0001	− 0.066	0.018	0.000
AD5	− 0.283	0.014	< .0001	− 0.293	0.015	< .0001	− 0.067	0.017	< .0001
Dimension ranking	PD-AD-MO-SC-UA			PD-AD-MO-SC-UA			MO-PD-SC-UA-AD		
21111	0.877			0.904			0.820		
12111	0.896			0.913			0.844		
11211	0.907			0.933			0.816		
11121	0.906			0.924			0.826		
11112	0.914			0.943			0.837		
55555	− 0.307			− 0.400			0.400		
No. of health states WTD, n (%)	271 (8.7)			412 (13.2)			0 (0.0)		

Number following dimension indicates level of severity (e.g., MO2 is Mobility level 2)

*cTTO* composite time trade-off, *MO* Mobility, *SC* Self-Care, *UA* Usual Activities, *PD* Pain/Discomfort, *AD* Anxiety/Depression, *WTD* worse-than-dead

^*^Denotes preference inversion

^Insignificant decrement from “no problems”

**Fig. 4:**
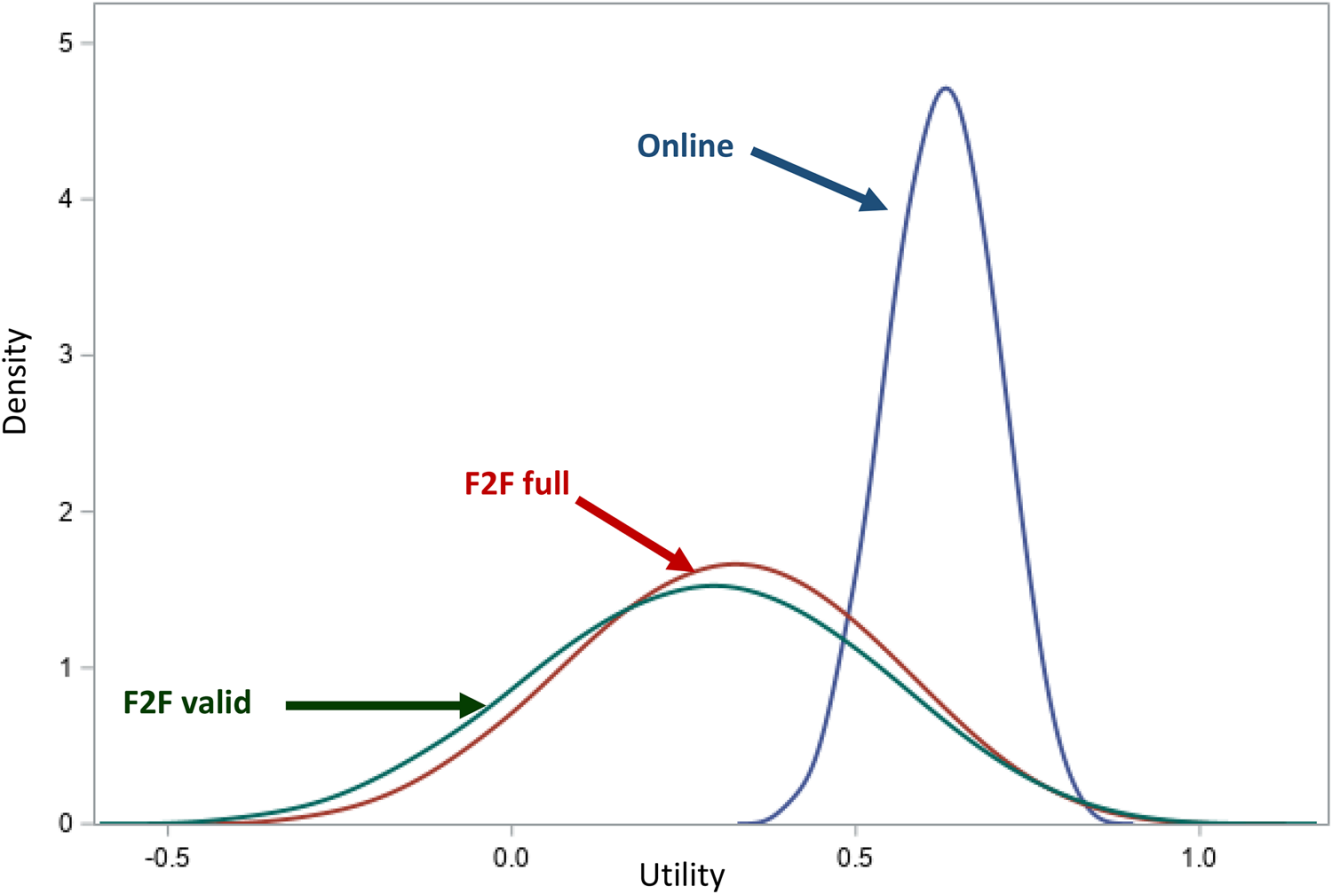
Kernel density plots for F2F Full, F2F Valid, and Online value sets based on linear regression with random intercept

The intercept for the online modeled value set was 0.846, whereas the intercepts for F2F_F_ and F2F_V_ were 0.963 and 0.993, respectively (Table 3). F2F_F_ and F2F_V_ value sets yielded 8.7% and 13.2% EQ-5D-5L health states as WTD, and the ranges of scale were 1.307 and 1.400, respectively (Table 3 and Fig. 4). In contrast, the online value set had no WTD health states as the value for 55555 was 0.400, and the range of scale was 0.446. All value set distributions were unimodal and approximately normally distributed (Fig. 4). The relative importance of dimensions differed drastically between F2F_F_/F2F_V_ and Online. The F2F_I_ value set had no WTD health states and shared a similar distribution to the Online value set.

In unadjusted, joint models of [1] F2F_F_ and Online and [2] F2F_V_ and Online responses, online data collection was associated with higher valuations of 0.31 and 0.34 utility units, respectively (Appendix P). After adjustment for respondent characteristics, the magnitude of valuation difference between modes remained relatively unchanged ([1] 0.27 and [2] 0.31). In joint F2F_I_ and Online models, the unadjusted and adjusted differences between comparators were 0.017 and 0.030 utility units, respectively (Online Appendix Q). After adjustment for respondent characteristics, the odds of at least 1 logical inconsistency was 3.635 times greater in online respondents compared to F2F_F_ (95% CI: 2.544–5.193).

## Discussion

The online sample reported poorer understanding of the TTO tasks, was less engaged with the tasks, and had poorer data validity compared to F2F Full and F2F Valid samples. In addition to the predetermined task engagement criteria such as number of trade-offs used, the online arm also had substantially greater portions of respondents who only traded in positive TTO values or did not trade any time. Although these response patterns were not invalid by definition, they demonstrated the unwillingness of online respondents to provide WTD values, possibly due to lack of understanding of the LT-TTO/WTD preference elicitation, decreased task engagement, and/or different underlying preference functions.

The validity of online elicited preferences was problematic, as demonstrated by the greater prevalence of inconsistencies. Online respondents were 13 times more likely to have at least 1 55555-inconsistency compared to F2F_V_ respondents. These 55555-involved inconsistencies were concerning as respondents should have noted 55555 was dominated by all other health states described by the EQ-5D-5L. Further, a smaller portion of online respondents indicated that the TTO task was easy to understand and these respondents did not provide more valid TTO values, leading to further concerns regarding the fidelity of the online TTO data.

The modeled value set of online responses had significant deficiencies even without appraisal against the F2F comparators. The value for the intercept (which can be interpreted as the value for 11111, a health state without any problems) was 0.846, far from the top of the utility scale. The value for 55555 was 0.400, meaning that online respondents felt that it was 0.400 utility units better-than-dead. Even if online respondents had systematically different preferences than face-to-face respondents, the resulting value set is difficult to justify from a validity standpoint.

The analyses using the F2F Invalid sample were not included as part of the primary analyses for several reasons. For some F2F_I_ respondents, interviewers completed a portion of the TTO tasks so the interview could proceed to less cognitively demanding tasks. Further, the small sample size (n = 72) meant that statistical testing may have been underpowered. However, if F2F_I_ is considered a group of heterogeneous, poor validity responses, its similarity to the online responses provides further evidence for the invalidity of online TTO preferences.

The TTO values were elicited from the general population. Comparatively, patients may provide preferences that are similarly valid in both online and F2F data collection because they may be more motivated and have greater insight into/experience with suboptimal health states, i.e., informedness. Longworth et al. used the cTTO to elicit preferences for Crohn’s disease outcomes from patients and general population respondents drawn from online panels [26]. Following exclusion of logically inconsistent results, the authors found that both the general population and patients provided valid utility values. Further analyses are necessary to determine how informedness affects TTO-based preferences elicited from online panel respondents.

Norman et al. also compared online and face-to-face TTO-based preference elicitation of EQ-5D health states, but the authors used a different TTO variant and randomized 107 respondents that were all recruited in-person [3]. Similar to this study, online responses had larger spikes at TTO values of 0 and 1. Dissimilar to this study, the Norman online cohort had a larger spike at −1, suggesting that the TTO values elicited may be sensitive to the TTO type and/or source of respondents.

This study was subject to several limitations. The effects of mode of administration/interviewer presence and source of respondents (i.e., online panel versus not) could not be separately estimated. However, this study provides evidence for a pragmatic, “comparative effectiveness” evaluation of real-world data preference elicitation, with face-to-face and online comparators representing typical recruitment and data collection methods of a given mode. A full factorial series of studies could help disentangle these separate influences [3]. Some performance differences between online and face-to-face may have been affected by the disparate sample sizes (e.g., number of insignificant utility decrements). As online responses were associated with more random error, a larger sample size may have been preferred in the online sample relative to the face-to-face sample. However, more online respondents may not contribute significantly to different measures of central tendency or other comparisons which do not depend on uncertainty in measurement, such as prevalence of inconsistent TTO valuations and mean elicited TTO values by misery score. As these benchmarks were quite dissimilar between F2F and online comparators, increasing the online sample size is unlikely to significantly affect how the modes compare. Adjusted models could not fully account for all respondent characteristics which can affect preferences, because they were unmeasured (e.g., personality) or because their measurement may be affected by social desirability bias (health/illness experience) [4, 27]. The differences in health/illness experience between modes is interesting, however, and further research should help address whether these are true differences due to selection pressures or reporting differences due to interviewer presence. Lastly, the extent to which the online approach to TTO data collection used in this study is generalizable is unclear, but the online platform was based on the EQ-VT and used the cTTO, both of which were informed by a robust program of research [20, 23, 28].

Although deploying a survey of TTO tasks to online, unsupervised respondents should likely not be the first choice for valuation studies, select methods of TTO or TTO-related implementation may succeed if other preference elicitation methods are deemed inadequate. For example, Devlin et al. proposed methods to estimate personal preference functions using simpler tasks [29]. If these tasks are administered within an online TTO survey, an assessment of whether TTO-based and task-based preferences match may help identify respondents who understood and engaged with the more cognitively challenging TTO tasks. Some ordinal tasks related to the TTO have also been developed, which could allow for utility estimation while retaining the TTO’s relative ease of analysis [30]. While data cleaning using predetermined or data-driven rules could isolate the most valid responses, caution must be applied as not to overly “curate” the data and inadvertently eliminate valid preferences which are external to the researchers’ chosen framework of valid preferences [10].

With greater understanding of mode and respondent source effects and ongoing TTO modifications, online preference elicitation of TTO values in the general population may be more viable in the future. However, the present approach to online TTO was unable to overcome possible issues with respondent engagement and task understanding.

## Electronic supplementary material

Below is the link to the electronic supplementary material.

Supplementary file1 (DOCX 1030 KB)

## Data Availability

Data and code may be available via request to corresponding author.
