# Supplementary material for: Comparison of online and face-to-face valuation of the EQ-5D-5L using composite time trade-off
Source: Qual Life Res. 2020 Nov 28;30(5):1433–44. doi: 10.1007/s11136-020-02712-1 (PMC8068705; doi:10.1007/s11136-020-02712-1)
Supplement: Supplementary file 1 — Supplementary file1 (DOCX 1030 KB) [file 11136_2020_2712_MOESM1_ESM.docx]

Appendix A – Visual presentations of Face-to-Face (top) and Online (bottom) composite time trade-off tasks
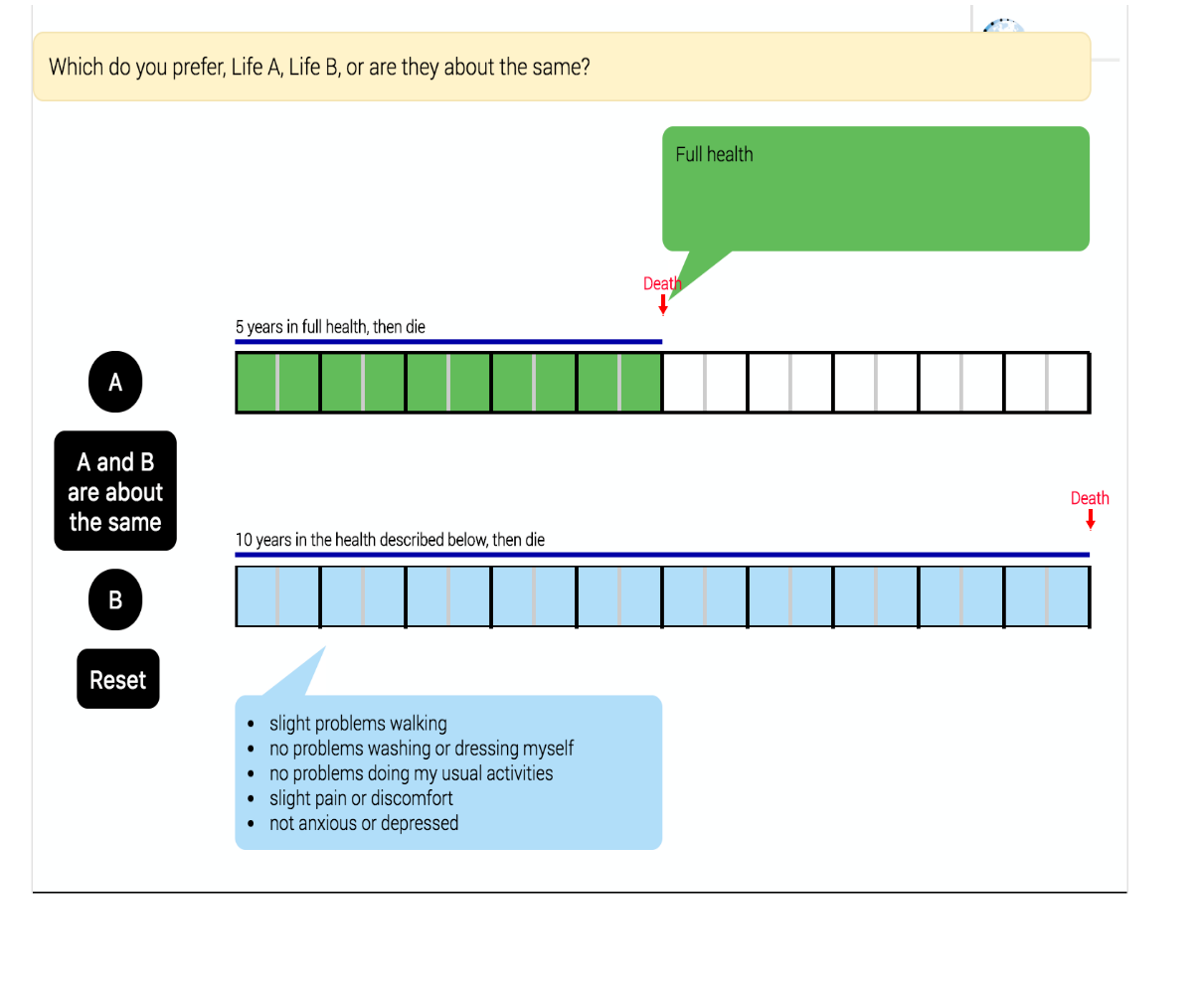


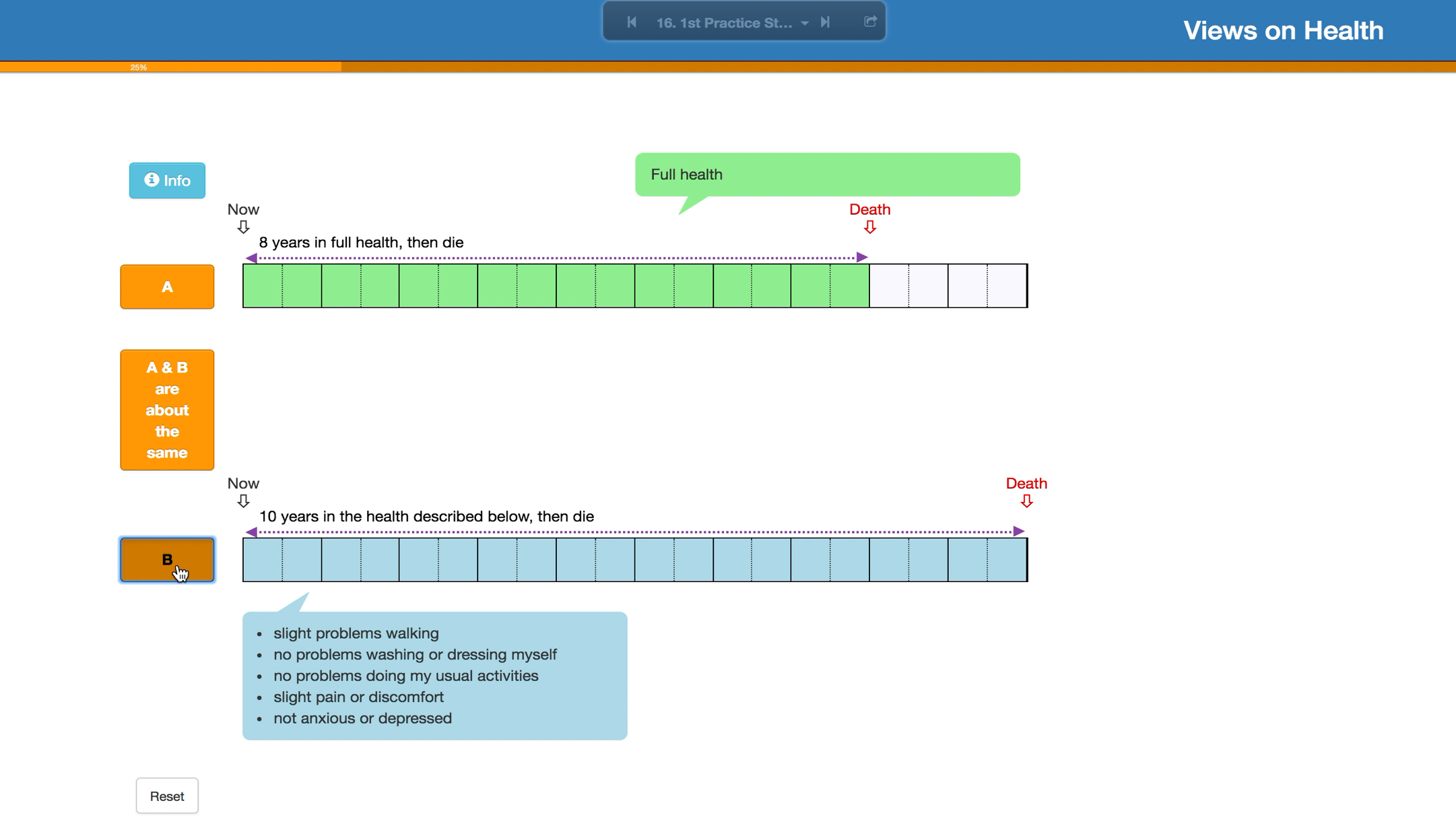


Appendix B – Changes from the face-to-face survey implemented in online data collection

| **Time trade-off task element** | **Changes implemented in online**  $\mathbf{✓}$ **if no change from F2F** |
| --- | --- |
| **Experimental design** | $✓$ |
| **Type of task** | $✓$ |
| **Number of tasks** | $✓$ (10 cTTO tasks) |
| **Minimum task time before reminder pop-up** | 15 seconds |
| **Health state presentation** | Read aloud and presented word by word to simulate F2F |
| **Example tasks** | EQ-5D-5L health state practice tasks not labeled as practice |
| **TTO Feedback module** | Removed due to difficulty explaining to online respondents |

Appendix C Automated ping-pong/titration process implemented in cTTO (adapted from Stolk 2019)

(Adapted from Stolk 2018, Value in Health). This figure illustrates the ping-pong/titration pattern of the cTTO implemented in the study according to the official EQ-VT routing implementation.

The x-axis represents the number of trade-offs, beginning with 0 for the initial task presentation of 10 years in Life A (perfect health) and 10 years in Life B (the suboptimal health state being valued). The y-axis represents the TTO value at each comparison of Life A and Life B. The colored arrows represent the TTO value change when the respondent preferred Life A (blue arrow) or Life B (green arrow) at a presented comparison.

For the engagement arm, respondents could not end the task before making at least 3 trade-offs. Four TTO values are possible if the respondent chooses to end the TTO task immediately following these 3 trade-offs: 0.6. 0.4, -0.4, and 0.6, as indicated by the orange line through the plot. If the respondent wished to assign TTO values of 0, 1, 0.5 or -0.5 to a health state, they would need to consciously continue trading until the TTO values returned to those values at trade-off 8.

Appendix D – Online health state presentation prior to the composite Time Trade-Off task
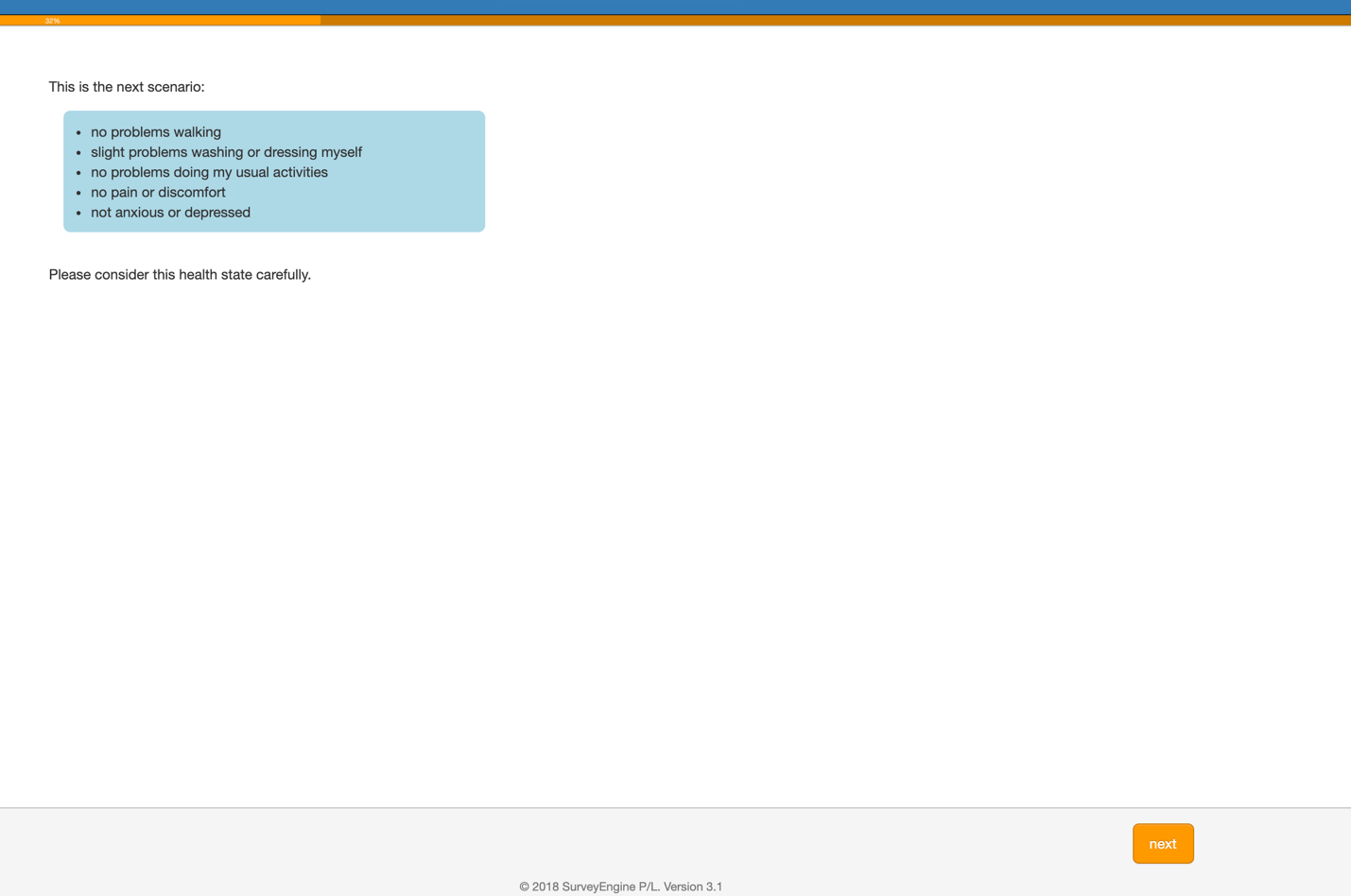


Appendix E – Online interactive wheelchair tutorial


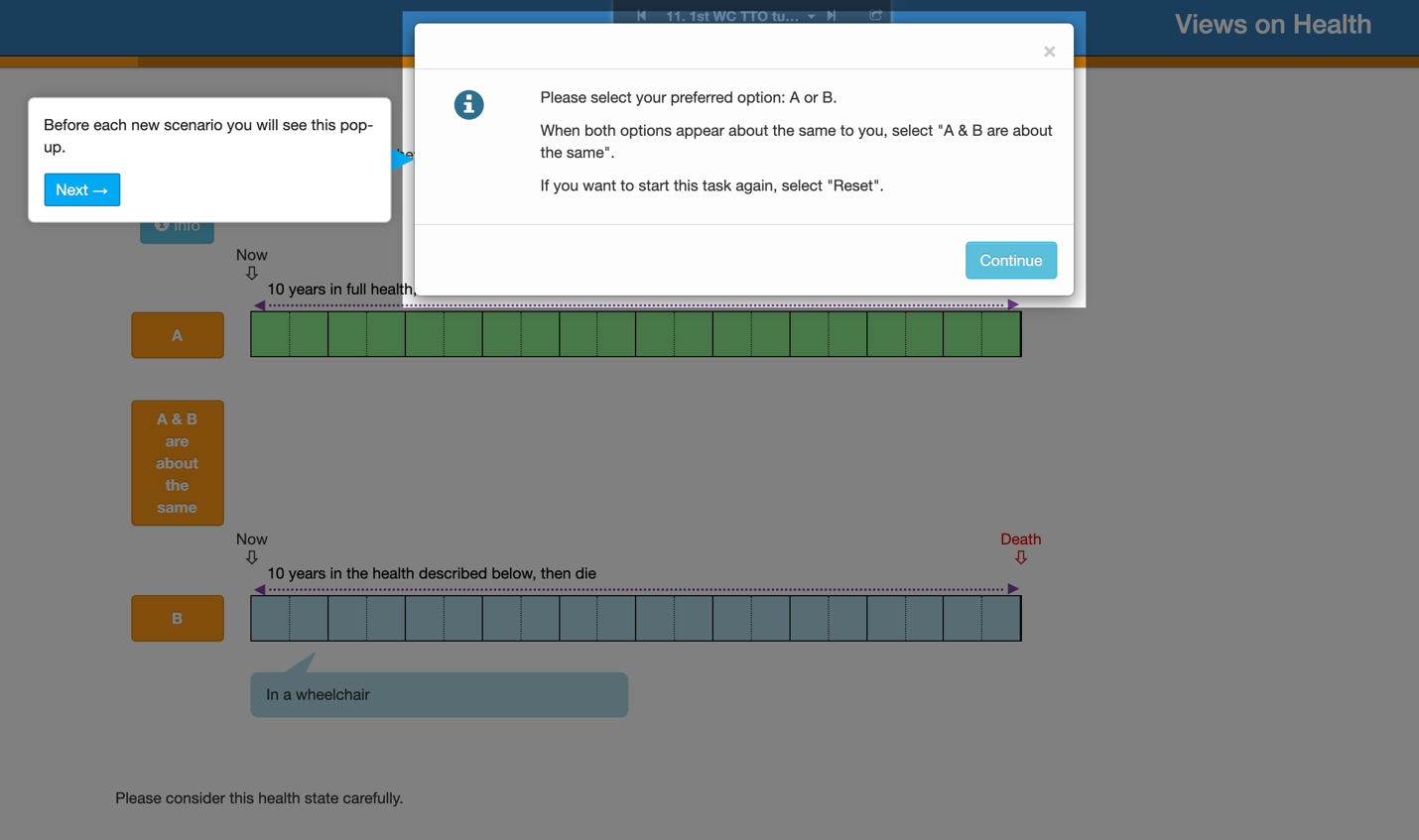


Appendix F – Respondents and responses included in the US EQ-5D-5L Face-to-Face valuation study and responses retained

**F2F Invalid cTTO responses**

n=720

**Respondents who did not understand cTTO**

**(F2F Invalid)**

n=72

**F2F Valid cTTO responses**

(n=9,386)

cTTO responses flagged by respondent during feedback module

n=1,234

**F2F respondents who understood cTTO**

**(F2F Valid)**

n=1,062

cTTO responses

(n=10,620)

**Face-to-face interviews**

**(F2F Full)**

n=1,134

**F2F Full cTTO responses**

n=11,340

Appendix G – Face-to-Face Invalid respondent characteristics

| Characteristic | (4)  F2F Invalid  N=72 | (3)  Online  N=501 |
| --- | --- | --- |
| Age, mean (SD), n (%) | 53.4 (18.2) | 45.9 (15.1) |
| 18-34 | 11 (15.3) | 149 (29.7) |
| 35-54 | 29 (40.3) | 180 (35.9) |
| 55+ | 32 (44.4) | 172 (34.3) |
| Range | 19 - 90 | 17-80 |
| Gender, n (%) |  |  |
| Male | 49 (68.1) | 251 (50.1) |
| Female | 23 (31.9) | 250 (49.9) |
| Gender, other (46) |  | -- |
| Race, n (%) |  |  |
| White | 24 (33.3) | 387 (77.3) |
| Black | 24 (33.3) | 63 (12.6) |
| Hispanic ethnicity, n (%) | 17 (23.6) | 75 (15.0) |
| Education level greater  than secondary, n (%) | 29 (40.3) | 344 (68.7) |
| Child dependents |  |  |
| None | 59 (81.9) | 338 (67.5) |
| Child(ren), ≤ 5 years old | 3 (4.1) | 65 (13.0) |
| Child(ren), 6 to 17 years old | 11 (15.3) | 138 (27.5) |
| Primary health insurance (47) |  |  |
| None | 9 (12.5) | 49 (9.8) |
| Public | 17 (23.6) | 204 (40.5) |
| Private | 46 (63.9) | 249 (49.7) |
| Country of birth, United States | 54 (74.0) | 475 (94.8) |
| History of illness, n (%) (45) |  |  |
| Hypertension | 25 (34.7) | 141 (28.1) |
| Arthritis | 23 (31.9) | 120 (24.0) |
| Diabetes | 16 (22.2) | 71 (14.2) |
| Heart Failure | 2 (2.8) | 11 (2.2) |
| Stroke | 3 (4.2) | 11 (2.2) |
| Bronchitis | 6 (8.3) | 18 (3.6) |
| Asthma | 7 (9.7) | 52 (10.4) |
| Depression | 25 (34.7) | 117 (23.4) |
| Migraine | 10 (13.9) | 58 (11.6) |
| Cancer | 6 (8.3) | 12 (2.4) |
| None | 16 (22.2) | 157 (31.3) |
| Health status, n (%) (44) |  |  |
| Excellent / Very good / Good | 57 (79.2) | 411 (82.0) |
| Fair / Poor | 15 (20.8) | 90 (18.0) |
| Self-reported EQ-VAS |  |  |
| Mean (SD) | 78.0 (17.2) | 73.6 (20.4) |
| Median (IQR) | 80 (20) | 80 (25) |
| Mobility |  |  |
| No problems | 45 (62.5) | 333 (66.5) |
| Slight problems | 14 (19.4) | 98 (19.6) |
| Some/Moderate problems | 6 (8.3) | 55 (11.0) |
| Severe problems | 5 (6.9) | 13 (2.6) |
| Unable to walk about | 2 (2.8) | 2 (0.4) |
| Self-care |  |  |
| No problems | 66 (91.7) | 420 (82.9) |
| Slight problems | 1 (1.4) | 50 (10.0) |
| Some/Moderate problems | 1 (1.4) | 24 (4.8) |
| Severe problems | 3 (4.2) | 5 (1.0) |
| Unable to wash or dress | 1 (1.4) | 2 (0.4) |
| Usual activities |  |  |
| No problems | 52 (72.2) | 328 (65.5) |
| Slight problems | 12 (16.7) | 103 (20.6) |
| Some/Moderate problems | 6 (8.3) | 54 (10.8) |
| Severe problems | 0 (0.0) | 13 (2.6) |
| Unable to do usual activities | 2 (2.8) | 3 (0.6) |
| Pain/Discomfort |  |  |
| No pain or discomfort | 33 (45.8) | 185 (36.9) |
| Slight pain or discomfort | 25 (34.7) | 172 (34.3) |
| Moderate pain or discomfort | 7 (9.7) | 104 (20.8 |
| Severe pain or discomfort | 3 (4.2) | 36 (7.2) |
| Extreme pain or discomfort | 4 (5.6) | 4 (0.8) |
| Anxiety/Depression |  |  |
| Not anxious or depressed | 44 (61.1) | 228 (45.5) |
| Slightly anxious or depressed | 17 (23.6) | 138 (27.5) |
| Moderately anxious or depressed | 8 (11.1) | 90 (18.0) |
| Severely anxious or depressed | 2 (2.8) | 29 (5.8) |
| Extremely anxious or depressed | 1 (1.4) | 16 (3.2) |
| "I found it easy to understand the questions I was asked" |  |  |
| Strongly agree | 32 (44.4) | 239 (47.7) |
| Agree | 17 (23.6) | 166 (33.13) |
| Neither agree nor disagree | 15 (20.8) | 50 (9.98) |
| Disagree | 4 (5.6) | 34 (6.79) |
| Strongly disagree | 4 (5.6) | 12 (2.4) |
| I found it easy to tell the difference between the lives I was asked to think about |  |  |
| Strongly agree | 22 (30.6) | 214 (42.71) |
| Agree | 24 (33.3) | 185 (36.93) |
| Neither agree nor disagree | 14 (19.4) | 60 (11.98) |
| Disagree | 6 (8.3) | 32 (6.39) |
| Strongly disagree | 6 (8.3) | 10 (2) |
| "I found it difficult to decide on my answers to the questions" |  |  |
| Strongly agree | 35 (48.6) | 90 (17.96) |
| Agree | 18 (25.0) | 144 (28.74) |
| Neither agree nor disagree | 13 (18.1) | 107 (21.36) |
| Disagree | 3 (4.2) | 92 (18.36) |
| Strongly disagree | 3 (4.2) | 68 (13.57) |

Appendix H Prevalence of tasks which used lag-time TTO and rate of conversion to WTD

|  | F2F Full | F2F Valid | F2F Invalid | Online |
| --- | --- | --- | --- | --- |
| % of tasks that used lag-time | 24.22% | 25.35% | 14.51% | 23.21% |
| % of tasks which used lag-time  and was assigned a WTD value | 93.77% | 94.62% | 81.72% | 37.10% |

F2F = Face-to-face

F2F = Face-to-face; Std dev = standard deviation

Appendix K Comparison of TTO task characteristics by trading behavior between F2F Full, F2F Valid, F2F Invalid, and Online samples

|  |  | F2F Full  (N=1134) | | | F2F Valid  (N=1062) | | | F2F Invalid  (N=72) | | | Online  (N=501) | | |
| --- | --- | --- | --- | --- | --- | --- | --- | --- | --- | --- | --- | --- | --- |
|  | Variable | Number of tasks | Mean (SD) | Range | Number of tasks | Mean (SD) | Range | Number of tasks | Mean (SD) | Range | Number of tasks | Mean (SD) | Range |
| BTD only traders | TTO moves | 3510 | 5.71 (4.69) | 1-107 | 2744 | 5.95 (4.4) | 1-107 | 380 | 2.87 (3.51) | 1-23 | 2320 | 5.98 (4.89) | 1-99 |
|  | TTO value | 3510 | 0.74 (0.28) | 0.05-1 | 2744 | 0.72 (0.29) | 0.05-1 | 380 | 0.91 (0.19) | 0.05-1 | 2320 | 0.79 (0.24) | 0.05-1 |
|  | TTO task time | 3510 | 56.32 (65.62) | 0.1-2115.14 | 2744 | 57.26 (55.17) | 0.26-932.17 | 380 | 38.4 (120.08) | 0.1-2115.14 | 2320 | 60.1 (63.33) | 20.27-1219.42 |
| BTD and WTD traders | TTO moves | 7830 | 7.03 (4.76) | 1-67 | 6642 | 7.06 (4.63) | 1-67 | 340 | 6 (7.07) | 1-55 | 2690 | 5.17 (6.4) | 1-89 |
|  | TTO value | 7830 | 0.14 (0.75) | -1-1 | 6642 | 0.12 (0.75) | -1-1 | 340 | 0.28 (0.7) | -1-1 | 2690 | 0.49 (0.5) | -1-1 |
|  | TTO task time | 7830 | 70.76 (64.06) | 0.21-1188.07 | 6642 | 69.94 (63.2) | 0.26-1188.07 | 340 | 73.38 (74.38) | 0.21-402.57 | 2690 | 66.11 (88.44) | 20.76-1540.84 |
| Non-traders  (All TTO values = 1) | TTO moves | 650 | 3.14 (5.46) | 1-107 | 390 | 4.32 (6.59) | 1-107 | 230 | 1.12 (0.99) | 1-11 | 360 | 2.1 (3.35) | 1-33 |
|  | TTO value | 650 | 1 (0) | 1-1 | 390 | 1 (0) | 1-1 | 230 | 1 (0) | 1-1 | 360 | 1 (0) | 1-1 |
|  | TTO task time | 650 | 34.14 (61.43) | 0.1-932.17 | 390 | 47.5 (70.3) | 0.26-932.17 | 230 | 8.93 (29.34) | 0.1-249.99 | 360 | 50.72 (74.14) | 20.27-1010.36 |
| All traders  (≥ 1 TTO value ≠ 1) | TTO moves | 10690 | 6.83 (4.65) | 1-89 | 8996 | 6.84 (4.45) | 1-67 | 490 | 5.87 (6.34) | 1-55 | 4650 | 5.81 (5.82) | 1-99 |
|  | TTO value | 10690 | 0.28 (0.7) | -1-1 | 8996 | 0.27 (0.71) | -1-1 | 490 | 0.43 (0.64) | -1-1 | 4650 | 0.6 (0.43) | -1-1 |
|  | TTO task time | 10690 | 68.25 (64.58) | 0.21-2115.14 | 8996 | 67.05 (60.68) | 0.26-1188.07 | 490 | 76.5 (116.6) | 0.21-2115.14 | 4650 | 64.3 (78.09) | 20.76-1540.84 |
| All tasks completed within 3 trade-offs | TTO moves | 420 | 1.05 (0.29) | 1-3 | 151 | 1.06 (0.26) | 1-3 | 250 | 1.04 (0.27) | 1-3 | 790 | 1.37 (0.59) | 1-3 |
|  | TTO value | 420 | 0.98 (0.13) | 0-1 | 151 | 0.98 (0.12) | 0-1 | 250 | 0.98 (0.14) | 0-1 | 790 | 0.7 (0.45) | -0.5-1 |
|  | TTO task time | 420 | 20.47 (44.25) | 0.1-661.21 | 151 | 38.13 (59.37) | 5.37-661.21 | 250 | 8.28 (26.55) | 0.1-249.99 | 790 | 51.94 (55.62) | 20.27-1010.36 |
| At least 1 task completed using 4 or more trade-offs | TTO moves | 10920 | 6.83 (4.74) | 1-107 | 9235 | 6.82 (4.57) | 1-107 | 470 | 6.11 (6.39) | 1-55 | 4220 | 6.33 (5.96) | 1-99 |
|  | TTO value | 10920 | 0.3 (0.7) | -1-1 | 9235 | 0.29 (0.71) | -1-1 | 470 | 0.41 (0.65) | -1-1 | 4220 | 0.61 (0.43) | -1-1 |
|  | TTO task time | 10920 | 68.05 (64.91) | 0.22-2115.14 | 9235 | 66.69 (61.16) | 0.26-1188.07 | 470 | 79.73 (118.19) | 0.22-2115.14 | 4220 | 65.46 (81.2) | 20.76-1540.84 |

Appendix L Percent of respondents with at least 1 inconsistency (any valued health state and involving 55555) delineated by sequence of 55555 presentation

|  | Inconsistency type by comparator | | | | | |
| --- | --- | --- | --- | --- | --- | --- |
|  | F2F Full | | F2F Valid | | Online | |
| Sequence of 55555 presentation | any health state | 55555 | any health state | 55555 | any health state | 55555 |
| First health state | 35.6% | 20.2% | 13.7% | 4.2% | 58.3% | 41.7% |
| First 3 health states | 31.1% | 14.7% | 14.4% | 3.4% | 60.3% | 44.2% |
| First 5 health states | 31.7% | 14.9% | 14.7% | 3.3% | 61.8% | 42.4% |
| Last 5 health states | 31.8% | 11.7% | 17.2% | 2.9% | 60.5% | 40.3% |
| Last 3 health states | 34.2% | 13.4% | 19.5% | 3.6% | 61.2% | 42.6% |
| Last health state | 30.2% | 10.3% | 20.0% | 4.5% | 60.0% | 36.0% |

Appendix M Bubble plots demonstrating number and magnitude of TTO inconsistency involving any valued health state (top) or 55555 (bottom) by comparator


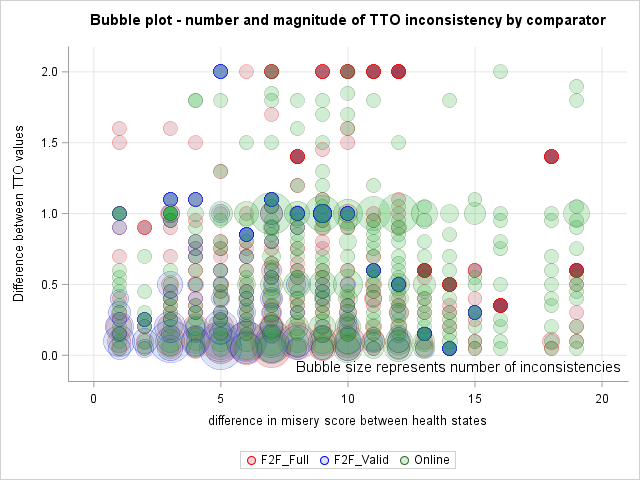


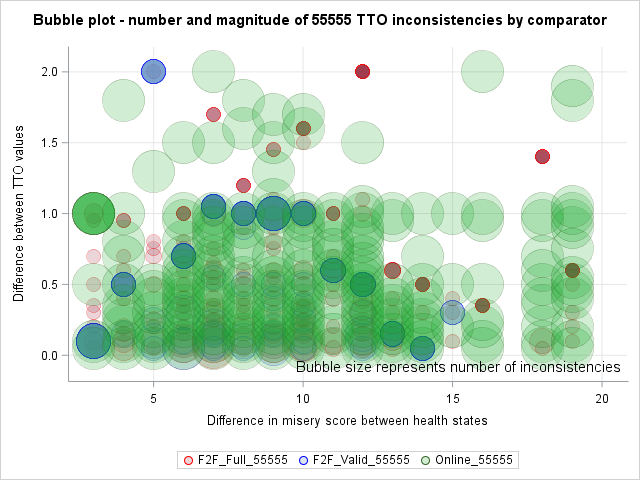


Bubble sizes are not to the same scale across plots and therefore cannot be directly compared

^

^

#p<0.05 ^p<0.001 for comparison to Online

Appendix O: Task and respondent-level engagement and validity measure comparisons for F2F_I_ compared to Online

|  |  | **(4)** | | **(3)** | | **(4) vs. (3) p-value** |
| --- | --- | --- | --- | --- | --- | --- |
|  |  | **F2F Invalid** | | **Online** | |  |
| **Task-level** |  | N=720 | | N=5010 | |  |
|  |  | Mean | SD |  | SD |  |
|  | Trade-offs | 4.35 | 5.70 | 5.55 | 5.76 | <0.001 |
|  | Time per task (s) | 54.92 | 102.54 | 63.33 | 77.88 | 0.035 |
|  | TTO value | 0.61 | 0.59 | 0.63 | 0.43 | 0.381 |
| **Respondent-level** |  | N=72 | | N=501 | |  |
|  |  | Mean | SD |  | SD |  |
|  | Number of inconsistencies | 2.18 | 3.23 | 2.77 | 3.68 | 0.156 |
|  | Number of 55555 inconsistencies | 1.14 | 2.08 | 1.26 | 2.04 | 0.639 |
|  | Time spent on survey(s) | 58.8 | 20.4 | 38.7 | 16.7 | <0.001 |

F2F = Face-to-face

Appendix P: Unadjusted and adjusted Face-to-Face Full and online and joint Face-to-Face Valid and Online models

|  |  | **Joint F2F (Full) and online cTTO model; unadjusted** | | |  | **Joint F2F (Full) and online cTTO model; adjusted** | | |  | **Joint F2F (valid) and online cTTO model; unadjusted** | | |  | **Joint F2F (valid) and online cTTO model; adjusted** | | |
| --- | --- | --- | --- | --- | --- | --- | --- | --- | --- | --- | --- | --- | --- | --- | --- | --- |
|  |  | Estimate | SE | p-value |  | Estimate | SE | p-value |  | Estimate | SE | p-value |  | Estimate | SE | p-value |
| Intercept |  | 0.832 | 0.017 | <.0001 |  | 0.944 | 0.074 | <.0001 |  | 0.830 | 0.017 | <.0001 |  | 0.956 | 0.074 | <.0001 |
| MO2 |  | -0.067 | 0.011 | <.0001 |  | -0.068 | 0.011 | <.0001 |  | -0.069 | 0.012 | <.0001 |  | -0.070 | 0.012 | <.0001 |
| MO3 |  | -0.098 | 0.011 | <.0001 |  | -0.099 | 0.012 | <.0001 |  | -0.099 | 0.012 | <.0001 |  | -0.100 | 0.012 | <.0001 |
| MO4 |  | -0.158 | 0.013 | <.0001 |  | -0.158 | 0.013 | <.0001 |  | -0.170 | 0.013 | <.0001 |  | -0.170 | 0.013 | <.0001 |
| MO5 |  | -0.210 | 0.011 | <.0001 |  | -0.211 | 0.012 | <.0001 |  | -0.226 | 0.012 | <.0001 |  | -0.228 | 0.012 | <.0001 |
| SC2 |  | -0.047 | 0.011 | <.0001 |  | -0.048 | 0.011 | <.0001 |  | -0.054 | 0.012 | <.0001 |  | -0.055 | 0.012 | <.0001 |
| SC3 |  | -0.078 | 0.012 | <.0001 |  | -0.079 | 0.012 | <.0001 |  | -0.087 | 0.013 | <.0001 |  | -0.088 | 0.013 | <.0001 |
| SC4 |  | -0.154 | 0.012 | <.0001 |  | -0.155 | 0.012 | <.0001 |  | -0.169 | 0.013 | <.0001 |  | -0.169 | 0.013 | <.0001 |
| SC5 |  | -0.169 | 0.011 | <.0001 |  | -0.170 | 0.011 | <.0001 |  | -0.177 | 0.012 | <.0001 |  | -0.177 | 0.012 | <.0001 |
| UA2 |  | -0.049 | 0.011 | <.0001 |  | -0.050 | 0.011 | <.0001 |  | -0.054 | 0.012 | <.0001 |  | -0.055 | 0.012 | <.0001 |
| UA3 |  | -0.082 | 0.012 | <.0001 |  | -0.083 | 0.012 | <.0001 |  | -0.089 | 0.013 | <.0001 |  | -0.091 | 0.013 | <.0001 |
| UA4 |  | -0.169 | 0.012 | <.0001 |  | -0.171 | 0.012 | <.0001 |  | -0.179 | 0.013 | <.0001 |  | -0.181 | 0.013 | <.0001 |
| UA5 |  | -0.153 | 0.011 | <.0001 |  | -0.153 | 0.011 | <.0001 |  | -0.167 | 0.012 | <.0001 |  | -0.167 | 0.012 | <.0001 |
| PD2 |  | -0.044 | 0.010 | <.0001 |  | -0.043 | 0.010 | <.0001 |  | -0.050 | 0.011 | <.0001 |  | -0.049 | 0.011 | <.0001 |
| PD3 |  | -0.072 | 0.012 | <.0001 |  | -0.071 | 0.012 | <.0001 |  | -0.075 | 0.013 | <.0001 |  | -0.074 | 0.013 | <.0001 |
| PD4 |  | -0.213 | 0.011 | <.0001 |  | -0.213 | 0.011 | <.0001 |  | -0.223 | 0.012 | <.0001 |  | -0.223 | 0.012 | <.0001 |
| PD5 |  | -0.264 | 0.012 | <.0001 |  | -0.265 | 0.012 | <.0001 |  | -0.274 | 0.013 | <.0001 |  | -0.276 | 0.013 | <.0001 |
| AD2 |  | -0.038 | 0.012 | 0.001 |  | -0.039 | 0.012 | 0.001 |  | -0.038 | 0.013 | 0.003 |  | -0.038 | 0.013 | 0.002 |
| AD3 |  | -0.092 | 0.013 | <.0001 |  | -0.093 | 0.013 | <.0001 |  | -0.096 | 0.014 | <.0001 |  | -0.097 | 0.014 | <.0001 |
| AD4 |  | -0.208 | 0.012 | <.0001 |  | -0.208 | 0.012 | <.0001 |  | -0.212 | 0.013 | <.0001 |  | -0.212 | 0.013 | <.0001 |
| AD5 |  | -0.219 | 0.011 | <.0001 |  | -0.220 | 0.011 | <.0001 |  | -0.217 | 0.012 | <.0001 |  | -0.218 | 0.012 | <.0001 |
| Online data collection  (Ref= F2F) |  | 0.306 | 0.022 | <.0001 |  | 0.261 | 0.036 | <.0001 |  | 0.336 | 0.022 | <.0001 |  | 0.306 | 0.037 | <.0001 |

MO: Mobility; SC: Self-Care; UA: Usual Activities; PD: Pain/Discomfort; AD: Anxiety/Depression; number following dimension indicates level of severity (e.g., MO2 is Mobility level 2); F2F: face-to-face

Appendix Q: Face-to-Face Invalid and online, separate and unadjusted and adjusted joint models

|  |  | **F2F Invalid (interviewer-judged invalid respondents only) cTTO** | | |  | **Online cTTO** | | |  | **Joint online and F2F (interviewer-judged invalid respondents only) model; unadjusted** | | |  | **Joint online and F2F (interviewer-judged invalid respondents only) model; adjusted** | | |
| --- | --- | --- | --- | --- | --- | --- | --- | --- | --- | --- | --- | --- | --- | --- | --- | --- |
|  |  | Estimate | SE | p-  value |  | Estimate | SE | p-  value |  | Estimate | SE | p-  value |  | Estimate | SE | p-  value |
| Intercept |  | 0.809 | 0.076 | <.0001 |  | 0.846 | 0.021 | <.0001 |  | 0.827 | 0.039 | <.0001 |  | 0.811 | 0.103 | <.0001 |
| MO2 |  | -0.088 | 0.050 | 0.079^ |  | -0.026 | 0.016 | 0.114^ |  | -0.033 | 0.016 | 0.032 |  | -0.036 | 0.016 | 0.023 |
| MO3 |  | -0.121 | 0.052 | 0.021 |  | -0.043 | 0.017 | 0.011 |  | -0.054 | 0.016 | 0.001 |  | -0.053 | 0.017 | 0.001 |
| MO4 |  | -0.087* | 0.058 | 0.136^ |  | -0.067 | 0.019 | 0.000 |  | -0.069 | 0.018 | <.0001 |  | -0.068 | 0.018 | <.0001 |
| MO5 |  | -0.126 | 0.054 | 0.019 |  | -0.112 | 0.017 | <.0001 |  | -0.114 | 0.016 | <.0001 |  | -0.115 | 0.016 | <.0001 |
| SC2 |  | -0.033 | 0.051 | 0.516^ |  | -0.003 | 0.016 | 0.874^ |  | -0.007 | 0.016 | 0.665 |  | -0.008 | 0.016 | 0.600 |
| SC3 |  | -0.022* | 0.056 | 0.691^ |  | -0.035 | 0.018 | 0.055^ |  | -0.033 | 0.017 | 0.060 |  | -0.032 | 0.018 | 0.072 |
| SC4 |  | 0.039* | 0.059 | 0.508^ |  | -0.098 | 0.018 | <.0001 |  | -0.083 | 0.017 | <.0001 |  | -0.084 | 0.018 | <.0001 |
| SC5 |  | -0.078 | 0.052 | 0.136^ |  | -0.077 | 0.016 | <.0001 |  | -0.077 | 0.016 | <.0001 |  | -0.076 | 0.016 | <.0001 |
| UA2 |  | 0.039* | 0.051 | 0.445^ |  | -0.030 | 0.017 | 0.075^ |  | -0.021 | 0.016 | 0.185 |  | -0.025 | 0.016 | 0.128 |
| UA3 |  | -0.067 | 0.057 | 0.242^ |  | -0.067 | 0.018 | 0.000 |  | -0.066 | 0.017 | <.0001 |  | -0.069 | 0.018 | <.0001 |
| UA4 |  | -0.010* | 0.055 | 0.859^ |  | -0.059 | 0.018 | 0.001 |  | -0.053 | 0.017 | 0.002 |  | -0.055 | 0.017 | 0.001 |
| UA5 |  | 0.037 | 0.050 | 0.455^ |  | -0.075 | 0.016 | <.0001 |  | -0.062 | 0.016 | <.0001 |  | -0.064 | 0.016 | <.0001 |
| PD2 |  | 0.014* | 0.047 | 0.769^ |  | -0.020 | 0.015 | 0.187^ |  | -0.017 | 0.015 | 0.256 |  | -0.017 | 0.015 | 0.261 |
| PD3 |  | 0.047* | 0.058 | 0.419^ |  | -0.023 | 0.018 | 0.210^ |  | -0.016 | 0.018 | 0.357 |  | -0.014 | 0.018 | 0.421 |
| PD4 |  | -0.029 | 0.049 | 0.556^ |  | -0.090 | 0.016 | <.0001 |  | -0.081 | 0.016 | <.0001 |  | -0.080 | 0.016 | <.0001 |
| PD5 |  | -0.131 | 0.057 | 0.021 |  | -0.108 | 0.018 | <.0001 |  | -0.110 | 0.017 | <.0001 |  | -0.110 | 0.017 | <.0001 |
| AD2 |  | -0.060 | 0.054 | 0.268^ |  | -0.010 | 0.018 | 0.586^ |  | -0.016 | 0.017 | 0.356 |  | -0.015 | 0.017 | 0.367 |
| AD3 |  | -0.108 | 0.060 | 0.075^ |  | -0.031 | 0.020 | 0.114^ |  | -0.042 | 0.019 | 0.026 |  | -0.041 | 0.019 | 0.031 |
| AD4 |  | -0.106* | 0.056 | 0.059^ |  | -0.066 | 0.018 | 0.000 |  | -0.070 | 0.017 | <.0001 |  | -0.067 | 0.017 | <.0001 |
| AD5 |  | -0.100* | 0.054 | 0.063^ |  | -0.067 | 0.017 | <.0001 |  | -0.072 | 0.016 | <.0001 |  | -0.072 | 0.016 | <.0001 |
| Online data collection  (Ref= F2F) |  | N/A | | |  | N/A | | |  | 0.017 | 0.038 | 0.651 |  | 0.030 | 0.050 | 0.548 |
| Dimension ranking |  | PD-MO-AD-SC-UA | | |  | MO-PD-SC-UA-AD | | |  | N/A | | |  | N/A | | |
| Estimated utility values by health state | | | | | |  | | |  |  | | |  |  | | |
| 21111 |  | 0.721 | | |  | 0.820 | | |  | N/A | | |  | N/A | | |
| 12111 |  | 0.776 | | |  | 0.844 | | |  |  |  |  |  |  |  |  |
| 11211 |  | 0.848 | | |  | 0.816 | | |  |  |  |  |  |  |  |  |
| 11121 |  | 0.823 | | |  | 0.826 | | |  |  |  |  |  |  |  |  |
| 11112 |  | 0.749 | | |  | 0.837 | | |  |  |  |  |  |  |  |  |
| No. of health states WTD  N (%) |  | 0 (0.0%) | | |  | 0 (0.0%) | | |  | N/A | | |  | N/A | | |

MO: Mobility; SC: Self-Care; UA: Usual Activities; PD: Pain/Discomfort; AD: Anxiety/Depression; number following dimension indicates level of severity (e.g., MO2 is Mobility level 2); F2F: face-to-face

*denotes preference inversion;

^insignificant decrement from “no problems”

Appendix R: Linear regression results for all comparators without respondent-level random effect

|  | **F2F Full** | | | **F2F Valid** | | | **F2F Invalid** | | | **Online** | | |
| --- | --- | --- | --- | --- | --- | --- | --- | --- | --- | --- | --- | --- |
|  | Value | SD | p-value | Value | SD | p-value | Value | SD | p-value | Value | SD | p-value |
| **Intercept** | 0.962 | 0.020 | <0.001 | 0.996 | 0.022 | <0.001 | 0.745 | 0.054 | <0.001 | 0.836 | 0.021 | <0.001 |
| **MO2** | -0.093 | 0.020 | <0.001 | -0.108 | 0.021 | <0.001 | -0.018 | 0.048 | 0.715 | -0.025 | 0.020 | 0.217 |
| **MO3** | -0.129 | 0.020 | <0.001 | -0.144 | 0.022 | <0.001 | -0.045 | 0.049 | 0.367 | -0.049 | 0.021 | 0.018 |
| **MO4** | -0.200 | 0.022 | <0.001 | -0.241 | 0.024 | <0.001 | -0.007 | 0.054 | 0.890 | -0.063 | 0.023 | 0.006 |
| **MO5** | -0.257 | 0.020 | <0.001 | -0.302 | 0.022 | <0.001 | -0.073 | 0.048 | 0.133 | -0.117 | 0.021 | <0.001 |
| **SC2** | -0.069 | 0.019 | <0.001 | -0.084 | 0.020 | <0.001 | 0.012 | 0.047 | 0.799 | 0.000 | 0.019 | 0.985 |
| **SC3** | -0.102 | 0.022 | <0.001 | -0.113 | 0.024 | <0.001 | -0.037 | 0.053 | 0.490 | -0.041 | 0.022 | 0.071 |
| **SC4** | -0.180 | 0.021 | <0.001 | -0.210 | 0.023 | <0.001 | -0.021 | 0.052 | 0.684 | -0.096 | 0.022 | <0.001 |
| **SC5** | -0.210 | 0.020 | <0.001 | -0.226 | 0.022 | <0.001 | -0.119 | 0.048 | 0.013 | -0.080 | 0.021 | <0.001 |
| **UA2** | -0.049 | 0.020 | 0.014 | -0.058 | 0.022 | 0.007 | 0.030 | 0.050 | 0.549 | -0.026 | 0.021 | 0.203 |
| **UA3** | -0.086 | 0.021 | <0.001 | -0.106 | 0.023 | <0.001 | 0.035 | 0.051 | 0.501 | -0.057 | 0.022 | 0.008 |
| **UA4** | -0.215 | 0.021 | <0.001 | -0.232 | 0.023 | <0.001 | -0.089 | 0.051 | 0.085 | -0.046 | 0.022 | 0.034 |
| **UA5** | -0.184 | 0.020 | <0.001 | -0.204 | 0.022 | <0.001 | -0.087 | 0.047 | 0.063 | -0.081 | 0.020 | <0.001 |
| **PD2** | -0.050 | 0.018 | 0.005 | -0.065 | 0.019 | 0.001 | 0.031 | 0.046 | 0.498 | -0.022 | 0.019 | 0.239 |
| **PD3** | -0.089 | 0.022 | <0.001 | -0.093 | 0.024 | <0.001 | -0.068 | 0.052 | 0.196 | -0.028 | 0.023 | 0.222 |
| **PD4** | -0.263 | 0.019 | <0.001 | -0.284 | 0.021 | <0.001 | -0.178 | 0.045 | 0.000 | -0.112 | 0.020 | <0.001 |
| **PD5** | -0.333 | 0.021 | <0.001 | -0.359 | 0.023 | <0.001 | -0.204 | 0.051 | 0.000 | -0.098 | 0.022 | <0.001 |
| **AD2** | -0.049 | 0.020 | 0.015 | -0.057 | 0.022 | 0.008 | -0.015 | 0.051 | 0.767 | 0.013 | 0.021 | 0.544 |
| **AD3** | -0.126 | 0.022 | <0.001 | -0.143 | 0.024 | <0.001 | -0.046 | 0.054 | 0.396 | -0.013 | 0.023 | 0.570 |
| **AD4** | -0.271 | 0.021 | <0.001 | -0.289 | 0.023 | <0.001 | -0.166 | 0.051 | 0.001 | -0.057 | 0.021 | 0.008 |
| **AD5** | -0.284 | 0.020 | <0.001 | -0.286 | 0.022 | <0.001 | -0.266 | 0.048 | <0.001 | -0.054 | 0.020 | 0.008 |

MO: Mobility; SC: Self-Care; UA: Usual Activities; PD: Pain/Discomfort; AD: Anxiety/Depression; number following dimension indicates level of severity (e.g., MO2 is Mobility level 2); F2F: face-to-face
